# The reality of “food porn”: Larger brain responses to food‐related cues than to erotic images predict cue‐induced eating

**DOI:** 10.1111/psyp.13309

**Published:** 2018-12-16

**Authors:** Francesco Versace, David W. Frank, Elise M. Stevens, Menton M. Deweese, Michele Guindani, Susan M. Schembre

**Affiliations:** ^1^ Department of Behavioral Science The University of Texas MD Anderson Cancer Center Houston Texas; ^2^ Oklahoma Tobacco Research Center The University of Oklahoma Health Sciences Center Oklahoma City Oklahoma; ^3^ Department of Teaching and Learning Vanderbilt University Nashville Tennessee; ^4^ Department of Statistics The University of California, Irvine Irvine California; ^5^ Department of Family and Community Medicine University of Arizona, College of Medicine–Tucson Tucson Arizona

**Keywords:** cue reactivity, endophenotypes, ERPs, incentive salience, late positive potential (LPP), sign tracking

## Abstract

While some individuals can defy the lure of temptation, many others find appetizing food irresistible. The goal of this study was to investigate the neuropsychological mechanisms that increase individuals' vulnerability to cue‐induced eating. Using ERPs, a direct measure of brain activity, we showed that individuals with larger late positive potentials in response to food‐related cues than to erotic images are more susceptible to cue‐induced eating and, in the presence of a palatable food option, eat more than twice as much as individuals with the opposite brain reactivity profile. By highlighting the presence of individual brain reactivity profiles associated with susceptibility to cue‐induced eating, these findings contribute to the understanding of the neurobiological basis of vulnerability to obesity.

## Abstract 2

Our findings contribute to the understanding of the biological bases underlying individual differences in vulnerability to cue‐induced eating, and they represent a step toward identifying new targets for personalized weight control interventions.

## 1 INTRODUCTION

Over 62,000 photos are shared worldwide each day under the hashtag #foodporn (Mejova, Abbar, & Haddadi, 2016). These images glamorize the highly palatable, high‐calorie foods that are believed to promote the maladaptive eating patterns contributing to today's obesity epidemic. A common assumption is that obese individuals have difficulty controlling food intake partly because food‐related stimuli elicit irresistible cravings by abnormally activating their brain's appetitive systems (Kenny, 2011b). Yet, studies comparing brain responses to food‐related cues in obese and lean individuals yielded inconsistent findings (Carbine et al., 2018; Field, Werthmann, & Franklin, 2016; Hendrikse et al., 2015; Versace & Schembre, 2015), suggesting that the neural underpinnings of human cue‐induced eating remain unclear. Given the multifactorial nature of a complex disease like obesity (Gortmaker et al., 2011), grouping study participants into categories based exclusively on body mass index (BMI) is likely to lump together individuals with substantially different characteristics, perhaps leading to the inconsistent findings reported in the literature. Endophenotypes (i.e., internal characteristics that mediate the relationship between genes and a given behavioral phenotype; Gottesman & Gould, 2003) have been proposed as an alternative to classifications based on external characteristics. To the extent to which endophenotypes reliably capture core features underlying behaviors such as compulsive cue‐induced eating, researchers can use them to create more homogenous subgroups and improve specificity of predictive analyses (Everitt & Robbins, 2016).

Following this strategy, we successfully used brain imaging techniques to identify candidate endophenotypes associated with elevated risk of smoking relapse during a quit attempt. Our findings consistently showed that smokers can be classified according to two brain reactivity profiles: one characterized by larger brain responses to cigarette‐related cues than pleasant stimuli (C > P) and the other characterized by larger brain responses to pleasant stimuli than cigarette‐related cues (P > C). Genetic analyses suggest that polymorphisms of genes influencing nAChR expression might be related to these neurophysiological profiles (Robinson et al., 2013). Importantly, these brain reactivity profiles are associated with differential risk of relapse: when smokers try to quit, individuals classified as C > P have significantly lower chances of achieving long‐term smoking abstinence than individuals classified as P > C (Versace et al., 2014, 2012; Versace, Claiborne, et al., 2017; Versace, Engelmann, et al., 2017). We interpreted these results in the light of those that emerged from preclinical studies aimed at identifying the neuropsychological underpinnings of cue‐induced compulsive behaviors (Flagel & Robinson, 2017; Sarter & Phillips, 2018). Specifically, measuring phasic dopamine responses during Pavlovian conditioning, Flagel et al. (2011) showed that animals that attribute incentive salience to cues predicting rewards are more vulnerable to compulsive cue‐induced behaviors, such as cue‐induced food seeking (Yager & Robinson, 2010) and cue‐induced drug seeking (Saunders & Robinson, 2013), than animals that do not attribute incentive salience to cues. Given the common mechanisms underlying drug addiction and obesity (Dileone, Taylor, & Picciotto, 2012; Kenny, 2011a; Volkow, Wang, Tomasi, & Baler, 2013), we hypothesized that, irrespective of BMI, individual differences in the tendency to attribute incentive salience to food‐related cues would underlie susceptibility to cue‐induced eating.

The first requisite to test our hypothesis is to measure the extent to which individuals attribute incentive salience to cues predicting food rewards. Incentive salience refers to the motivational properties that make a stimulus wanted (Berridge & Robinson, 2016). Motivation can be defined as what makes an organism work to obtain rewards or to avoid punishments (Pessoa, 2009). By appropriately and efficiently attributing incentive salience to stimuli predicting rewards, organisms can prioritize, modify, and adapt their consummatory behaviors to the ever‐changing environment (Di Chiara, 2002). Stimuli with high incentive salience capture attention, activate affective states, and motivate behaviors (Berridge, 2012). The incentive salience of biologically meaningful rewards (e.g., food, sex) and the stimuli that predict them is coded by dopamine signals in the mesocorticolimbic systems. Midbrain dopaminergic signals can enhance the representation of reward‐related stimuli in the cortical visual systems (Hickey & Peelen, 2015). Importantly, the cortical activity generated in the visual systems by affectively charged stimuli can be directly and noninvasively assessed with high temporal accuracy using ERPs, specifically by measuring the amplitude of the late positive potential (LPP; Cuthbert, Schupp, Bradley, Birbaumer, & Lang, 2000; Keil et al., 2002; Lang & Bradley, 2010; Liu, Huang, McGinnis‐Deweese, Keil, & Ding, 2012; Sabatinelli, Lang, Keil, & Bradley, 2007; Schupp et al., 2000; Versace et al., 2011). The LPP is considered the most reliable neurophysiological index of the extent to which visual stimuli engage the motivational brain circuits that guide adaptive perceptual and motor processes (Bradley, 2009). Both pleasant and unpleasant contents increase the amplitude of the LPP over central and parietal sites as a function of their emotional arousal (e.g., LPP to erotica and mutilations > LPP to romantic and sad > LPP to neutral images; Minnix et al., 2013; Schupp et al., 2004; Weinberg & Hajcak, 2010). The affective modulation of the LPP is present for both unconditioned and conditioned stimuli (Bacigalupo & Luck, 2018), it has high temporal stability (Codispoti, Ferrari, & Bradley, 2007), and it is resistant to manipulations affecting stimuli's perceptual composition (Bradley, Hamby, Löw, & Lang, 2007; De Cesarei & Codispoti, 2006), exposure time (Codispoti, Mazzetti, & Bradley, 2009), and repetition (Deweese, Codispoti, Robinson, Cinciripini, & Versace, 2018; Ferrari, Codispoti, & Bradley, 2017). These characteristics make the LPP a good measure to estimate the level of incentive salience that individuals attribute to cues predicting food rewards.

The second requisite to test our hypothesis is to classify individuals based on their tendency to attribute incentive salience to cues predicting food rewards. A classification based only on the differences between LPPs evoked by food‐related cues and neutral stimuli would not be appropriate because the responses evoked by nonfood‐related emotional stimuli must be taken into account to appropriately scale and interpret the cue‐minus‐neutral LPP difference (Oliver, Jentink, Drobes, & Evans, 2016; Versace, Engelmann, et al., 2017; Versace & Schembre, 2015). To avoid this pitfall, we proposed cluster analysis as a data‐driven classification approach capable of taking into account the LPP responses evoked by more than two contents. Cluster analysis is a multivariate technique that classifies objects (i.e., participants) based on their characteristics (Hair & Black, 2000). In previous studies, when we applied *k*‐means cluster analysis to the LPP responses evoked by emotional images and cues predicting rewards (such as food or nicotine), the algorithm reliably identified two groups of individuals: one characterized by larger LPPs to the cues than to pleasant stimuli, the other by larger LPPs to the pleasant stimuli than to the cues (Engelmann, Versace, Gewirtz, & Cinciripini, 2016; Versace et al., 2014, 2012; Versace, Kypriotakis, Basen‐Engquist, & Schembre, 2016). These findings show that by applying *k*‐means cluster analysis to the LPP responses evoked by a wide array of images it is possible to identify endophenotypes associated with individual differences in the tendency to attribute incentive salience to cues predicting rewards. However, our previous studies were limited in that we did not directly measure the extent to which these brain reactivity profiles underlie susceptibility to cue‐induced behaviors such as cue‐induced eating.

In the current study, we aimed to address this limitation by measuring brain responses as well as eating behavior during a cued food delivery task (Deweese et al., 2015). We hypothesized that individuals attributing higher levels of incentive salience to cues predicting food rewards would be more susceptible to cue‐induced eating than individuals attributing lower levels of incentive salience to cues predicting food rewards.

## 2 METHOD

### 2.1 Participants

We recruited 60 individuals from the Houston metropolitan area using flyers and magazine and newspaper advertisements. Participants were eligible for the study if they were between 18 and 65 years of age, were neither pregnant nor breastfeeding, and did not report any history of psychiatric disorders, seizures, head injuries with loss of consciousness, uncorrected visual impairments, eating disorders, allergies, or diet‐related chronic diseases that might have prevented consumption of M&M's chocolate candies. All participants received monetary compensation for their time and for parking/travel, totaling $60. Due to poor recording quality, largely attributed to excessive movement during the task, 11 participants were excluded at various stages of the data reduction process (see below), leaving 49 participants in the final sample (aged 24–65 years, 45% female; 41% overweight, 37% obese). Table 1 shows the sample characteristics. A preliminary power analysis indicated that a sample size of 20 individuals per group would ensure at least 80% power to detect a moderate effect size (Cohen's *d*) of 0.46 or higher in a two‐tailed *t* test, corresponding to a mean difference of 4.6 chocolate candies between the two groups (assuming a conservative estimate of standard deviation = 10, alpha = 0.05). To take data losses into account, we planned to enroll a total of 60 participants and stopped recruitment once we reached this goal. All data analyses were performed once recruitment ended.

**Table 1:** Participant demographic information and questionnaire scores by cluster membership

Characteristic	All (*N = *49)	C > P (*N = *20)	P > C (*N = *29)	*p* value
Age (years)	47	46	48	0.46
Women	45%	35%	51%	0.25
Race				
African American	67%	75%	62%	
Caucasian	26%	20%	31%	
Other	7%	5%	7%	
BMI	31	31	31	
BIS				
Attentional	14.16	15.75	13.07	0.01
Motor	21.49	22.00	21.14	0.49
Nonplanning	23.06	25.15	21.62	0.04
CESD	7.20	8.25	6.48	0.24
SHAPS	47.55	48.15	47.14	0.53
PANAS+	34.63	35.35	34.14	0.65
PANAS−	17.39	19.10	16.21	0.18
WREQ				
Routine restraint	1.76	1.58	1.88	0.27
Compensatory restraint	2.09	1.98	2.16	0.33
Susceptibility to external cues	1.94	2.09	1.83	0.55
Emotional eating	1.60	1.64	1.57	0.72
SLIM (pre‐)	−4.43	−8.97	−1.29	0.45
SLIM (post‐)	−10.15	−14.88	−6.89	0.50

Note

*p* values estimated by independent *t* tests or chi‐square analyses. The SLIM was used to assess appetite before the beginning (pre‐) and after the end of the experiment (post‐). C > P = individuals with larger LPPs to cues predicting food than to erotic stimuli; P > C = individuals with larger LPPs to erotic stimuli than to cues predicting food; BMI = body mass index; BIS = Barratt Impulsiveness Scale; CESD = The Center for Epidemiological Studies Depression Scale; SHAPS = Snaith‐Hamilton Pleasure Scale; PANAS = Positive and Negative Affect Schedule; WREQ = Weight‐Related Eating Questionnaire; SLIM = Satiety Labeled Intensity Magnitude.

### 2.2 Procedures

The study included a telephone interview to verify study eligibility, followed by one in‐person laboratory visit. At the in‐person visit, a research assistant reviewed the study with the participant and obtained written informed consent. Then, the research assistant measured the participant's height and weight and, using a computer‐assisted procedure, collected answers to a series of questionnaires about impulsivity, mood, hedonic capacity, eating behaviors, and hunger. At the completion of the questionnaire assessment, the research assistant placed the sensors for the EEG recording and provided the participant with detailed task instructions. The research assistant then left the room and started the EEG recording. At the end of the session, the research assistant removed the sensors, debriefed, and compensated the participant. All study procedures were approved by the University of Texas MD Anderson Cancer Center Institutional Review Board.

### 2.3 Materials

#### 2.3.1 Questionnaires

The computerized battery included the following questionnaires:

Barratt Impulsiveness Scale (BIS). The BIS (Patton, Stanford, & Barratt, 1995) includes 30 items describing common impulsive or nonimpulsive behaviors and preferences designed to assess the personality/behavioral construct of impulsiveness.

Center for Epidemiological Studies Depression Scale (CES‐D) (brief). The brief version of the CES‐D (Andresen, Malmgren, Carter, & Patrick, 1993) is a 10‐item self‐report instrument assessing the frequency of several depressive symptoms and originally developed for studying depressive symptomatology in the general population.

Snaith‐Hamilton Pleasure Scale (SHAPS). The SHAPS (Snaith et al., 1995) is a self‐report measure of anhedonia that, unlike other instruments, was specifically developed to be unaffected by social class, gender, age, dietary habits, or nationality. The SHAPS is a reliable and valid questionnaire designed to assess hedonic tone in patient and nonpatient populations (Franken, Rassin, & Muris, 2007).

Positive and Negative Affect Schedule (PANAS). The PANAS (Watson, Clark, & Tellegen, 1988) is a 20‐item self‐report instrument designed to measure the two primary measures of mood: positive and negative affect. This instrument is a reliable and valid measure of the two mood constructs (Crawford & Henry, 2004).

Weight‐Related Eating Questionnaire (WREQ). The 16‐item WREQ (Schembre, Greene, & Melanson, 2009) assesses four theory‐based aspects of eating behavior labeled compensatory restraint, routine restraint, susceptibility to external cues, and emotional eating.

Satiety Labeled Intensity Magnitude (SLIM). The SLIM (Cardello, Schutz, Lesher, & Merrill, 2005) is a sensitive, reliable, and easy to‐use scale for measuring perceived satiety. Participants completed the SLIM before and after the cued food delivery task using a paper and pencil version of the questionnaire.

#### 2.3.2 Cued food delivery task

During the cued food delivery task, participants viewed a series of images presented with a computer using E‐Prime software (version 2.0.8.74; PST Inc., Pittsburgh, PA) on a 17‐inch LCD monitor. The chocolate candies were delivered in a receptacle within arm's reach from the participant, situated to the right of the computer monitor (Deweese et al., 2015). The images belonged to eight categories covering a variety of content: neutral (people involved in mundane activities, household objects), highly arousing pleasant (erotica) and unpleasant (mutilations), low arousing pleasant (romantic) and unpleasant (violence), unpleasant objects (pollution), and palatable food (sweet, savory). The images were selected from the International Affective Picture System (Lang, Bradley, & Cuthbert, 2008) and from a database of images that we used in previous studies (Versace et al., 2016).

The task was divided into six equivalent blocks lasting approximately 5 min each. Every block consisted of a pseudorandom (i.e., no more than two consecutive images of the same category) presentation of 55 images: 10 neutral, 10 pleasant (5 erotica, 5 romantic), 15 unpleasant (5 mutilations, 5 violence, 5 pollution), and 20 food‐related (10 sweet, 10 savory). Images were not repeated during the task. One category of food images (either sweet or savory, counterbalanced across subjects) was designated as the “food‐paired” category: 1,000 ms after image onset, a chocolate candy was released from a dispenser and, through a tube, was delivered in a receptacle where the participant could pick it up and either eat it or deposit it in a box. The food‐paired image remained visible on the screen until the participant either pushed a button to indicate having consumed the candy or until the candy was dropped in the deposit box. All other images, including the “food‐unpaired” images (i.e., images of food not followed by candy delivery), were presented for 2,200 ms. A random intertrial interval of 500–2,000 ms separated each trial. Instructions at the beginning of the task indicated to the participant which food category was designated as the food predictive category. In this way, the participant did not have to learn the contingency, and all trials could be used in the analyses. To ensure that the participant fully understood the instructions, we ran 11 test trials, two of which were followed by the delivery of a candy.

### 2.4 EEG acquisition

During the task, we continuously recorded the EEG using a 129‐channel Geodesic Sensor Net, amplified with an AC‐coupled high input impedance (200 MΩ) amplifier (Geodesic EEG System 200, Electrical Geodesics Inc., Eugene, OR) and referenced to Cz. The sampling rate was 250 Hz, and data were filtered online by using 0.1 Hz high‐pass and 100 Hz low‐pass filters. Scalp impedance of each sensor was kept below 50 KΩ, as suggested by the manufacturer.

### 2.5 Data reduction

After EEG collection, we filtered the data with a 30‐Hz low‐pass filter, inspected the EEG traces to evaluate the quality of the recording, and identified and interpolated (using spherical splines) channels contaminated by artifacts for more than 50% of the recording time. At this stage, we discarded three participants due to poor quality of the EEG recording. For the remaining 57 participants, we corrected eyeblinks using a spatial filtering method as implemented in BESA version 5.1.8.10 (MEGIS Software GmbH, Gräfelfing, Germany). We transformed the EEG data to the average reference and segmented the data using the picture onset to time‐lock the ERPs. The segments started 100 ms before picture onset and ended 1,100 ms later. Baseline was defined as the 100‐ms interval preceding picture onset. Artifacts affecting sensors within each trial were identified using the following criteria: EEG amplitude above 100 or below –100 μV, absolute voltage difference between any two data points within the segment larger than 100 μV, voltage difference between two contiguous data points above 25 μV, and less than 0.5 μV variation for more than 100 ms. A segment was excluded from the subsequent averages if more than 10% of the sensors within the segment were contaminated by artifacts. At the end of this process, the average ERPs were calculated at each scalp site for each picture category. Participants were excluded from the analyses if they had fewer than 20% of the possible trials included in any category average (eight participants were excluded at this stage). The final sample included 49 participants.

### 2.6 LPP

**Figure 1:**
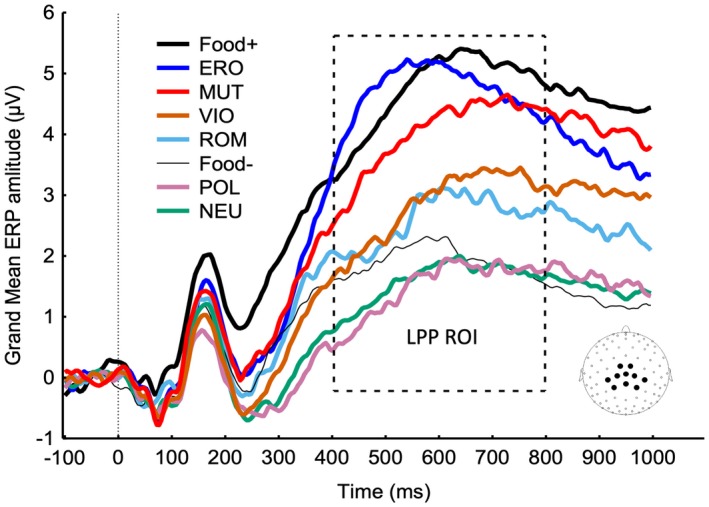
ERPs from centroparietal sites (see inset for electrode location) show that, on average, both pleasant and unpleasant contents increase the amplitude of the late positive potential (LPP) as a function of their emotional arousal. The box highlights the time region of interest (ROI) used to compute the LPP amplitude for each stimulus category. Food+ = food images paired with food delivery; Food− = food images not paired with food delivery; ERO = erotica; ROM = romantic; NEU = neutral; POL = pollution; VIO = violence; MUT = mutilations

The amplitude of the LPP evoked by each picture category was our measure of incentive salience. We calculated the LPP for each picture category for each participant by averaging the voltage recorded between 400 and 800 ms after picture onset from 10 central and parietal sensors (EGI HydroCel Geodesic Sensor Net sensors: 7, 31, 37, 54, 55, 79, 80, 87, 106, 129; see Figure 1 inset). This group of sensors, also used in our previous studies (Minnix et al., 2013; Versace et al., 2016, 2012), covers the area where the LPP amplitude differences between neutral and emotional pictures is maximal. A preliminary analysis aimed at confirming that the amplitude of the LPP increased as a function of the emotional arousal of the stimuli.

### 2.7 Classification of participants

To classify participants, we followed the same procedure that we used in our previous studies (Engelmann et al., 2016; Versace et al., 2016, 2012). For each individual, we calculated the mean LPP evoked by each stimulus category (i.e., food paired, erotica, romantic, food unpaired, neutral, pollution, violence, mutilations) between 400 and 800 ms over 10 centroparietal sensors. The eight LPP values calculated for each individual were entered into the cluster analysis. To account for individual variation in absolute voltage amplitude, we standardized the LPP values using ipsatization (Hicks, 1970). Then, we classified individuals based on their brain reactivity profiles using *k*‐means cluster analysis as implemented in the R statistical package (R Core Team, 2014). Cluster analysis is a data‐driven multivariate technique that groups individuals by minimizing within‐group variability and maximizing between‐groups variability (Hair & Black, 2000). The algorithm is unsupervised, using as constraints only the number of clusters and the variables used for deriving the solution. We assessed the optimal number of clusters and the corresponding classification using the Silhouette coefficient method (Rousseeuw, 1987) and the gap statistics (Tibshirani, Walther, & Hastie, 2001). It is important to note that the groups extracted using cluster analysis can differ with respect to any brain reactivity pattern; hence, the first analytic steps consisted of a series of validation checks aimed at confirming (a) the reliability of using the amplitude of the LPP to measure the emotional arousal of the nonfood‐related visual stimuli used in the experiment, and (b) the replicability of the categorization based on the LPP responses.

### 2.8 Statistical analyses

#### 2.8.1 ERPs

The first validation check tested whether both groups extracted using *k*‐means cluster analysis showed increasingly larger LPPs for images with increasing emotional arousal (i.e., erotica and mutilations > romantic and violence > neutral and pollution). Within each group, we tested the presence of a quadratic trend using polynomial contrasts. The second validation check tested whether the two brain reactivity profiles extracted using cluster analysis fit the hypothesized dichotomy (i.e., one group showing larger LPPs to food‐predictive images than to pleasant images, and the other group showing the opposite pattern) and whether the two groups differed in reactivity to any image category. To conduct these tests, we ran an analysis of variance (ANOVA) using the amplitude of the LPP as the dependent variable, the eight picture categories (food‐predictive, erotica, romantic, food‐nonpredictive, neutral, pollution, violence, mutilations) as a within‐subject factor, and the two groups as a between‐subjects factor. To account for violations of sphericity, we used multivariate ANOVA (Vasey & Thayer, 1987). To test for the presence of significant differences among categories within and between groups, we used pairwise comparisons with Bonferroni correction.

#### 2.8.2 Cue‐induced eating

To test for the presence of statistically significant between‐groups differences in the number of chocolate candies eaten by each participant during the experiment, we used the nonparametric Mann–Whitney *U* test. Then, to take into account overdispersion in the data, we also assessed the statistical significance of the differences in eating behavior in the two groups using a quasi‐Poisson generalized linear regression model with a scale dispersion parameter. Finally, we adjusted the analysis for the influence of potential confounding variables on eating behavior, by considering age, gender, BMI, and (pre‐experiment) level of appetite as additional covariates in the Poisson generalized linear regression model.

#### 2.8.3 Self‐report questionnaires and demographics

To test whether the two groups identified by the cluster analysis differed in age, gender, BMI, impulsivity, and mood, we conducted ANOVAs on these variables. The self‐reported level of satiety before and after the session was compared between the two groups using ANOVA and, for each group, we tested whether there was a significant difference from the *neither hungry nor full* anchor point before and after the session.

## 3 RESULTS

### 3.1 LPP

Figure 1 shows the ERP waveforms for each image category (i.e., food‐paired, erotica, romantic, food‐unpaired, neutral, pollution, violence, mutilations) averaged across the whole sample. As expected, the mean amplitude of the LPP increased as a function of the images' emotional arousal (Bonferroni‐corrected pairwise comparisons on the LPP responses showed that erotica and mutilations > romantic and violence > neutral contents; all *p < *0.0001). Furthermore, the LPP evoked by food‐paired images was significantly larger than the LPP evoked by food‐unpaired images (Bonferroni‐corrected *p < *0.0001). The LPP evoked by food‐unpaired images was not significantly different than the LPP evoked by neutral stimuli.

### 3.2 Classification of participants

**Figure 2:**
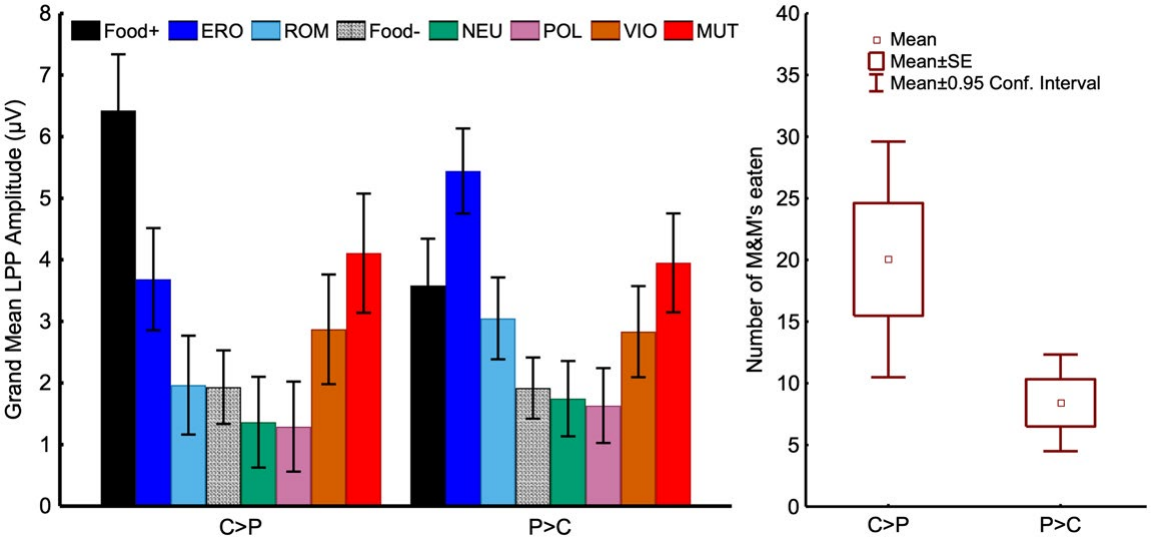
Left: The *k*‐means cluster analysis performed on the LPP responses yielded two clusters fitting with the hypothesized dichotomy. Some individuals (C > P, *N = *20) reacted more to Food+ images than to erotic images (*p < *0.0001), while others (P > C, *N = *29) had the opposite brain reactivity pattern (*p < *0.0001). The two groups had comparable LPP responses to all categories of stimuli except to food‐paired images (*p < *0.0001). The between‐groups difference observed for erotic stimuli was not significant after applying Bonferroni correction (*p = *0.15). Right: Individuals classified as C > P ate more than twice as many chocolate candies as individuals classified as P > C (20 vs. 8; *U = *188.5, *p = *0.036). Food+ = food images paired with food delivery; Food− = food images not paired with food delivery; ERO = erotica; ROM = romantic; NEU = neutral; POL = pollution; VIO = violence; MUT = mutilations; LPP = late positive potential. Error bars represent 0.95 confidence intervals

To classify participants, we applied *k*‐means cluster analysis to their LPP responses. The results of the gap statistic method and the silhouette method (online supporting information Figure S1 and S2), as implemented in the R module factoextra (Kassambara & Mundt, 2017) confirmed that a two‐cluster solution was the most appropriate to describe the underlying structure of the data. Figure 2 (left panel) shows that, in line with our hypotheses, one group (*N = *20, 41% of the sample) had larger LPPs to food‐paired cues than to erotic images (*p < *0.0001), whereas the other group (*N = *29, 59% of the sample) had larger LPPs to erotic images than to food‐paired cues (*p < *0.0001). To highlight the continuity between the results observed here and those from our previous studies, where we found that differences in brain reactivity to pleasant stimuli (P) and reward‐related cues (C) predicted compulsive nicotine use, we decided to label the two groups identified here as C > P and P > C. Both groups showed the typical reactivity pattern such that, irrespective of hedonic content, the amplitude of the LPP increased as a function of the images' emotional arousal. Excluding food images, the quadratic trend was significant for both groups (*p < *0.0001). The two groups had comparable LPP responses to all categories of stimuli except to food‐paired images (*p < *0.0001). Also, the between‐groups difference observed for erotic stimuli was not significant after applying Bonferroni correction (*p = *0.15). The two groups also had similar demographic characteristics and level of hunger at the beginning of the experiment (Table 1). The pattern of results and the proportion of individuals assigned to the two clusters were similar regardless of whether sweet or savory contents predicted food delivery. In summary, these results indicate that, although every participant was aware that food‐paired stimuli predicted food delivery, some individuals (C > P) attributed more incentive salience to food‐paired images than to erotic images, while others (P > C) processed food‐paired stimuli as though they had low incentive salience.

### 3.3 Cue‐induced eating

Figure 2 (right panel) shows that individuals classified as C > P ate more than twice as many chocolate candies as individuals classified as P > C (20 vs. 8; *U = *188.5, *p = *0.036). In the quasi‐Poisson, individuals classified as C > P ate chocolate candies at a rate that was 2.2 times greater (95% CI: 1.14, 4.37; *p = *0.024) than that of individuals classified as P > C, after adjusting for potential confounders (age, BMI, gender, and pre‐experiment hunger level; see supporting information Appendix S1 Results and Tables S2 and S3). The number of candies eaten by individuals classified as P > C or C > P was similar regardless of whether sweet or savory content preceded food delivery (8 vs. 21 and 8 vs. 18, respectively).

## 4 DISCUSSION

This experiment aimed at testing the hypothesis that individual differences in the tendency to attribute incentive salience to cues predicting rewards underlie vulnerability to cue‐induced behaviors. To test this hypothesis, we measured ERPs to a wide array of affectively charged visual stimuli and the number of chocolate candies eaten during a cued food delivery task. Applying a multivariate data‐driven classification approach to the brain responses evoked by the visual stimuli presented during the task, we identified two brain reactivity profiles that predicted cue‐induced eating: individuals with larger LPPs to cues predicting food delivery than to erotic images ate more than twice as many chocolate candies than individuals with larger LPPs to erotic images than cues predicting food delivery.

We believe that the two brain reactivity profiles identified in this experiment represent candidate endophenotypes that might correspond to those observed in rodents, where larger phasic dopamine responses to stimuli predicting rewards than to actual rewards in the nucleus accumbens are associated with vulnerability to cue‐induced compulsive behaviors (Flagel et al., 2011). In animals, dopamine responses to cues predicting rewards encode the level of incentive salience that individuals attribute to these stimuli (Berridge, 2007; Berridge & Robinson, 1998). fMRI studies in humans showed that midbrain dopaminergic activity mediates the visual representation of reward‐related stimuli in the visual cortex (Hickey & Peelen, 2015). Accordingly, the amplitude of the LPP, an ERP component generated in the occipital‐parietal cortex (Keil et al., 2002; Sabatinelli et al., 2007), reliably indexes the extent to which visual stimuli engage the motivational brain circuits that guide adaptive perceptual and motor processes (Bradley, 2009; Lang & Bradley, 2010; Lang, Bradley, & Cuthbert, 1997; Minnix et al., 2013; Olofsson, Nordin, Sequeira, & Polich, 2008; Schupp et al., 2004; Weinberg & Hajcak, 2010). Therefore, in line with what animal models indicate, the findings from our study support the hypothesis that individuals with the tendency to attribute high levels of incentive salience to cues predicting food rewards are more prone to cue‐induced eating.

One potential interpretative ambiguity is the fact that both pleasant and unpleasant stimuli increase the amplitude of the LPP. However, we think that it is extremely unlikely that the results reported here are the consequence of individuals finding erotic or food‐related stimuli unpleasant or threatening. Nevertheless, we are adapting the experimental paradigm used here to the fMRI environment to more precisely define the brain networks responsible for the differences observed here. Future studies should also determine the extent to which the brain reactivity profiles that we identified here meet all the criteria to be defined as an endophenotype (i.e., heritability, state independency, presence in the unaffected relatives of cases; Gottesman & Gould, 2003). Until all the necessary evidence is collected, the brain reactivity profiles identified here should only be considered candidate endophenotypes.

To classify participants, we used *k*‐means cluster analysis. *K*‐means cluster analysis is a widely used data‐driven multivariate algorithm that classifies individuals into a specified number of clusters based on their characteristics (i.e., the individual LPP reactivity profiles). Importantly, the algorithm is unsupervised, since it does not use any external information to guide how individuals should be grouped. The outcomes of our previous studies (Engelmann et al., 2016; Versace et al., 2014, 2016, 2012) led us to hypothesize a two‐cluster solution as the most appropriate to describe the underlying structure of the data. Our hypothesis was confirmed by both the silhouette and the gap statistic methods. Importantly, the pattern of the Cluster × Picture Category interaction replicates what we observed in our previous studies: the main feature extracted by the algorithm is related to individual differences in reactivity to cues predicting food rewards, relative to other nonfood‐related pleasant stimuli. In fact, while reactivity to unpleasant stimuli varied significantly both within and between groups, all individuals classified as P > C showed larger LPPs to erotic stimuli than to food‐related cues, and only one individual in the C > P group did not show larger responses to food‐related than to erotic stimuli. The *k*‐means algorithm, however, also has some limitations. One is that it can be used to classify individuals only after the complete sample has been collected. This feature limits the possibility to use it in clinical settings, where individuals should be classified on a subject‐by‐subject basis to effectively use the classification outcomes to tailor treatments (Cinciripini et al., 2017). To overcome this limitation, we are currently testing the extent to which discriminant functions (Duarte Silva & Stam, 1995) can be used to classify individuals on a subject‐by‐subject basis to then tailor treatments to the specific characteristics of the patient (Versace, Claiborne, et al., 2017). Another limitation of the *k*‐means algorithm is that it does not provide information about the uncertainty of the classification outcomes. It is likely that the tendency to attribute incentive salience to cues predicting rewards varies along a continuum, rather than being an all‐or‐none dichotomous variable. Furthermore, in this experiment we did not test the stability of the classification outcome across multiple sessions. Results from preclinical studies have shown that some individuals might oscillate between different classifications (Meyer et al., 2012). In light of these findings, future studies should aim at developing a classification algorithm that will take into account both the stability of the classification across multiple sessions and the level of uncertainty associated with the classification of each individual.

One advantage of our experimental paradigm is the possibility to let participants know at the beginning of the experiment which stimulus category predicts food delivery. Previous studies already showed that emotionally arousing naturalistic images (including reward‐related cues) can be used as reinforcers in second‐order conditioning paradigms (Deweese, Robinson, Cinciripini, & Versace, 2016; Littel & Franken, 2012) and that task‐irrelevant Pavlovian cues can influence human behavior (Garofalo & di Pellegrino, 2015). Therefore, by ensuring that all participants are aware of the cue‐food contingency, this paradigm eliminates the need for carrying out preliminary training sessions and allows for all artifact‐free trials to be included in the statistical analyses. Furthermore, by using naturalistic images rather than arbitrary visual patterns to signal impending food delivery, we preserved the face validity of the task. Additionally, by including two categories of food images (i.e., sweet, savory) and counterbalancing across subjects the category that signals food delivery, we effectively tested the extent to which individuals attribute incentive salience to the same stimuli when they do or do not predict actual rewards. We can think about the two conditions that we created in our experiment as approximations of what usually happens in everyday life, where fast food logos reliably indicate that food is available inside the store displaying the logo. This is in opposition to what often occurs in human research laboratories, where the presentation of food images reliably indicates that food is not available until the end of the experiment. Unsurprisingly, our results show that, in general, individuals attribute more incentive salience to food‐related cues when they signal impending food availability than when they do not. Another important feature of our experimental paradigm is the inclusion of a wide array of visual stimuli that differed in both valence and emotional arousal. Preclinical studies showed that the representation of value in the brain's valuation system adapts to the range of values available at any given time (Padoa‐Schioppa, 2009). Hence, including a wide array of emotional categories is necessary to accurately assess the incentive salience attributed to cues predicting rewards (Oliver et al., 2016; Versace, Engelmann, et al., 2017; Versace & Schembre, 2015). The large LPP difference that we observed between the predictive and the nonpredictive cues in this experiment suggests that researchers interested in understanding the role of cues as triggers of compulsive eating should consider how the availability of rewards during the experiment and the presence of other emotionally arousing stimuli might impact participants' neurobehavioral responses to the cues.

This experimental paradigm might contribute to better characterizing the neuropsychological underpinnings of other disorders where poor impulse control plays a significant role. Preclinical studies showed that animals attributing incentive salience to cues predicting food rewards also have the tendency to attribute incentive salience to cues predicting drug delivery, a trait that makes these animals more vulnerable to addictionlike behaviors (e.g., cue‐induced drug self‐administration and drug self‐administration reinstatement after extinction; Saunders & Robinson, 2013; Tomie, Grimes, & Pohorecky, 2008; Tunstall & Kearns, 2015). Results obtained from smokers (Engelmann et al., 2016; Mahler & de Wit, 2010; Versace et al., 2014, 2012) suggest that the tendency to attribute incentive salience to cues predicting rewards might also underlie multiple maladaptive behaviors in humans. We (Versace et al., 2014, 2012; Versace, Claiborne, et al., 2017) have found that smokers with larger brain responses to cigarette‐related cues than non‐nicotine‐related pleasant stimuli are more prone to relapse than smokers with larger brain responses to pleasant stimuli than to cigarette‐related cues. Future studies should investigate the extent to which the neuropsychological profiles identified here map onto those hypothesized to underlie “sign‐tracking” and “goal‐tracking” behaviors in animals (Flagel & Robinson, 2017; Sarter & Phillips, 2018; Tomie et al., 2008). Sign‐ and goal‐tracking behaviors emerge during Pavlovian conditioning, when a discrete cue (the sign) predicts the delivery of a food reward (the goal) at a different location. Under these circumstances, when the cue appears, some animals (referred to as sign trackers) tend to approach it, while others (referred to as goal trackers) tend to approach the location where the food will be delivered. Studies that investigated the neuropsychological underpinnings of these behaviors in animal models showed that sign tracking is the consequence of the animal's tendency to attribute incentive salience to cues predicting rewards (Flagel et al., 2011).

Another aspect that should be investigated in more detail in future studies is the relationship between the endophenotypes identified here and impulsivity. The results from the BIS (Patton et al., 1995) indicate that individuals classified as C > P report slightly higher scores on the attentional and nonplanning impulsivity subscales than individuals classified as P > C. Results from animal models showed that rats prone to attribute incentive salience to reward‐predictive cues are also more prone to impulsive actions (Lovic, Saunders, Yager, & Robinson, 2011). In line with these results, Garofalo and di Pellegrino (2015) showed that, in humans, individuals categorized as sign trackers report lower impulse control than goal trackers. Replicating our findings using an objective measure of impulsive action should contribute to determining the extent to which results from animal and human models converge regarding the role that impulsivity has in increasing vulnerability to cue‐induced behaviors.

One potential application of this paradigm might be to rapidly test the efficacy of treatments aimed at improving the ability to resist cue‐induced behaviors before starting large‐scale clinical trials. Another clinical application would be to use this paradigm to assess vulnerability to addictive behaviors in at‐risk individuals without exposing them to any substance of abuse (e.g., before opioids are prescribed for the first time or in drug‐naïve young individuals).

To conclude, while several factors motivate food consumption, our findings show that the tendency to attribute more incentive salience to food‐related cues than to nonfood‐related rewards significantly increases the likelihood of an individual to engage in maladaptive eating behaviors. By contributing to the understanding of the biological basis underlying individual differences in vulnerability to cue‐induced eating, our findings represent a step toward identifying new targets for personalized weight control interventions aimed at regulating the intense motivation to eat that many individuals experience in the presence of cues associated with highly palatable foods.

## Supporting information


**Figure S1**
**Figure S2**
**Table S1**
**Table S2**
**Table S3**

## References

[psyp13309-bib-0001] Andresen, E. M. , Malmgren, J. A. , Carter, W. B. , & Patrick, D. L. (1993). Screening for depression in well older adults: Evaluation of a short form of the CES‐D (Center for Epidemiologic Studies Depression Scale). American Journal of Preventive Medicine, 10(2), 77–84. 10.4236/health.2013.53A078 8037935

[psyp13309-bib-0002] Bacigalupo, F. , & Luck, S. J. (2018). Event‐related potential components as measures of aversive conditioning in humans. Psychophysiology, 55(4), e13015 10.1111/psyp.13015 PMC5849492 28949030

[psyp13309-bib-0003] Berridge, K. C. (2007). The debate over dopamine's role in reward: The case for incentive salience. Psychopharmacology, 191(3), 391–431. 10.1007/s00213-006-0578-x 17072591

[psyp13309-bib-0004] Berridge, K. C. (2012). From prediction error to incentive salience: Mesolimbic computation of reward motivation. European Journal of Neuroscience, 35(7), 1124–1143. 10.1111/j.1460-9568.2012.07990.x 22487042 PMC3325516

[psyp13309-bib-0005] Berridge, K. C. , & Robinson, T. E. (1998). What is the role of dopamine in reward: Hedonic impact, reward learning, or incentive salience? Brain Research Reviews, 28(3), 309–369. 10.1016/S0165-0173(98)00019-8 9858756

[psyp13309-bib-0006] Berridge, K. C. , & Robinson, T. E. (2016). Liking, wanting, and the incentive‐sensitization theory of addiction. American Psychologist, 71(8), 670–679. 10.1037/amp0000059 27977239 PMC5171207

[psyp13309-bib-0007] Bradley, M. M. (2009). Natural selective attention: Orienting and emotion. Psychophysiology, 46(1), 1–11. 10.1111/j.1469-8986.2008.00702.x 18778317 PMC3645482

[psyp13309-bib-0008] Bradley, M. M. , Hamby, S. , Löw, A. , & Lang, P. J. (2007). Brain potentials in perception: Picture complexity and emotional arousal. Psychophysiology, 44(3), 364–373. 10.1111/j.1469-8986.2007.00520.x 17433095

[psyp13309-bib-0009] Carbine, K. A. , Rodeback, R. , Modersitzki, E. , Miner, M. , Lecheminant, J. D. , & Larson, M. J. (2018). The utility of event‐related potentials (ERPs) in understanding food‐related cognition: A systematic review and recommendations. Appetite, 128, 58–78. 10.1016/j.appet.2018.05.135 29787830

[psyp13309-bib-0010] Cardello, A. V. , Schutz, H. G. , Lesher, L. L. , & Merrill, E. (2005). Development and testing of a labeled magnitude scale of perceived satiety. Appetite, 44(1), 1–13. 10.1016/j.appet.2004.05.007 15604029

[psyp13309-bib-0011] Cinciripini, P. M. , Green, C. E. , Robinson, J. D. , Karam‐Hage, M. A. , Engelmann, J. M. , Minnix, J. A. , … Versace, F. (2017). Benefits of varenicline vs. bupropion for smoking cessation: A Bayesian analysis of the interaction of reward sensitivity and treatment. Psychopharmacology, 234(11), 1769–1779. 10.1007/s00213-017-4580-2 28275830 PMC5901731

[psyp13309-bib-0012] Codispoti, M. , Ferrari, V. , & Bradley, M. M. (2007). Repetition and event‐related potentials: Distinguishing early and late processes in affective picture perception. Journal of Cognitive Neuroscience, 19(4), 577–586. 10.1162/jocn.2007.19.4.577 17381249

[psyp13309-bib-0013] Codispoti, M. , Mazzetti, M. , & Bradley, M. M. (2009). Unmasking emotion: Exposure duration and emotional engagement. Psychophysiology, 46(4), 731–738. 10.1111/j.1469-8986.2009.00804.x 19386053

[psyp13309-bib-0014] Crawford, J. R. , & Henry, J. D. (2004). The positive and negative affect schedule (PANAS): Construct validity, measurement properties and normative data in a large non‐clinical sample. British Journal of Clinical Psychology, 43(Pt 3), 245–265. 10.1348/0144665031752934 15333231

[psyp13309-bib-0015] Cuthbert, B. N. , Schupp, H. T. , Bradley, M. M. , Birbaumer, N. , & Lang, P. J. (2000). Brain potentials in affective picture processing: Covariation with autonomic arousal and affective report. Biological Psychology, 52(2), 95–111. 10.1016/S0301-0511(99)00044-7 10699350

[psyp13309-bib-0016] De Cesarei, A. , & Codispoti, M. (2006). When does size not matter? Effects of stimulus size on affective modulation. Psychophysiology, 43(2), 207–215. 10.1111/j.1469-8986.2006.00392.x 16712591

[psyp13309-bib-0017] Deweese, M. M. , Claiborne, K. N. , Ng, J. , Dirba, D. D. , Stewart, H. L. , Schembre, S. M. , & Versace, F. (2015). Dispensing apparatus for use in a cued food delivery task. MethodsX, 2, 446–457. 10.1016/j.mex.2015.11.002 26870667 PMC4678323

[psyp13309-bib-0018] Deweese, M. M. , Codispoti, M. , Robinson, J. D. , Cinciripini, P. M. , & Versace, F. (2018). Cigarette cues capture attention of smokers and never‐smokers, but for different reasons. Drug and Alcohol Dependence, 185(Feb), 50–57. 10.1016/j.drugalcdep.2017.12.010 29427915 PMC5889726

[psyp13309-bib-0019] Deweese, M. , Robinson, J. D. , Cinciripini, P. , & Versace, F. (2016). Conditioned cortical reactivity to cues predicting cigarette‐related or pleasant images. International Journal of Psychophysiology, 101, 59–68. 10.1016/j.ijpsycho.2016.01.007 26826400 PMC4853025

[psyp13309-bib-0020] Di Chiara, G. (2002). Nucleus accumbens shell and core dopamine: Differential role in behavior and addictions. Behavioural Brain Research, 137(1), 75–114. 10.1016/S0166-4328(02)00286-3 12445717

[psyp13309-bib-0021] Dileone, R. J. , Taylor, J. R. , & Picciotto, M. R. (2012). The drive to eat: Comparisons and distinctions between mechanisms of food reward and drug addiction. Nature Neuroscience, 15(10), 1330–1335. 10.1038/nn.3202 23007187 PMC3570269

[psyp13309-bib-0022] Duarte Silva, A. P. , & Stam, A. (1995). Discriminant analysis In Grimm L. G. & Yarnold P. R. (Eds.), Reading and understanding more multivariate statistics (pp. 277–318). Washington, DC: American Psychological Association.

[psyp13309-bib-0023] Engelmann, J. M. , Versace, F. , Gewirtz, J. C. , & Cinciripini, P. M. (2016). Individual differences in brain responses to cigarette‐related cues and pleasant stimuli in young smokers. Drug and Alcohol Dependence, 163, 229–235. 10.1016/j.drugalcdep.2016.04.025 27141838 PMC4880545

[psyp13309-bib-0024] Everitt, B. J. , & Robbins, T. W. (2016). Drug addiction: Updating actions to habits to compulsions ten years on. Annual Review of Psychology, 67(1), 150807174122003 10.1146/annurev-psych-122414-033457 26253543

[psyp13309-bib-0025] Ferrari, V. , Codispoti, M. , & Bradley, M. M. (2017). Repetition and ERPs during emotional scene processing: A selective review. International Journal of Psychophysiology, 111, 170–177. 10.1016/j.ijpsycho.2016.07.496 27418540

[psyp13309-bib-0026] Field, M. , Werthmann, J. , & Franken, I. H. A. (2016). The role of attentional bias in obesity and addiction. Health Psychology, 35(8), 767–780. 10.1017/CBO9781107415324.004 27505196

[psyp13309-bib-0027] Flagel, S. B. , Clark, J. J. , Robinson, T. E. , Mayo, L. , Czuj, A. , Willuhn, I. , … Akil, H. (2011). A selective role for dopamine in stimulus‐reward learning. Nature, 469(7328), 53–57. 10.1038/nature09588 21150898 PMC3058375

[psyp13309-bib-0028] Flagel, S. B. , & Robinson, T. E. (2017). Neurobiological basis of individual variation in stimulus‐reward learning. Current Opinion in Behavioral Sciences, 13, 178–185. 10.1016/j.cobeha.2016.12.004 28670608 PMC5486979

[psyp13309-bib-0029] Franken, I. H. A. , Rassin, E. , & Muris, P. (2007). The assessment of anhedonia in clinical and non‐clinical populations: Further validation of the Snaith‐Hamilton Pleasure Scale (SHAPS). Journal of Affective Disorders, 99, 83–89. 10.1016/j.jad.2006.08.020 16996138

[psyp13309-bib-0030] Garofalo, S. , & di Pellegrino, G. (2015). Individual differences in the influence of task‐irrelevant Pavlovian cues on human behavior. Frontiers in Behavioral Neuroscience, 9(Jun), 163 10.3389/fnbeh.2015.00163 26157371 PMC4478391

[psyp13309-bib-0031] Gortmaker, S. L. , Swinburn, B. A. , Levy, D. , Carter, R. , Mabry, P. L. , Finegood, D. T. , … Moodie, M. L. (2011). Changing the future of obesity: Science, policy, and action. Lancet, 378(9793), 838–847. 10.1016/S0140-6736(11)60815-5 21872752 PMC3417037

[psyp13309-bib-0032] Gottesman, I. I. , & Gould, T. D. (2003). The endophenotype concept in psychiatry: Etymology and strategic intentions. Amercian Journal of Psychiatry, 160(Apr), 636–645. 10.1176/appi.ajp.160.4.636 12668349

[psyp13309-bib-0033] Hair, J. F. , & Black, W. C. (2000). Cluster analysis In Grimm L. G. & Yarnold P. R. (Eds.), Reading and understanding more multivariate statistics (pp. 147–206). Washington, DC: American Psychological Association.

[psyp13309-bib-0034] Hendrikse, J. J. , Cachia, R. L. , Kothe, E. J. , McPhie, S. , Skouteris, H. , & Hayden, M. J. (2015). Attentional biases for food cues in overweight and individuals with obesity: A systematic review of the literature. Obesity Reviews, 16, 424–432. 10.1111/obr.12265 25752592

[psyp13309-bib-0035] Hickey, C. , & Peelen, M. V. (2015). Neural mechanisms of incentive salience in naturalistic human vision. Neuron, 85(3), 512–518. 10.1016/j.neuron.2014.12.049 25654257

[psyp13309-bib-0036] Hicks, L. E. (1970). Some properties of ipsative, normative, and forced‐choice normative measures. Psychological Bulletin, 74(3), 167–184. 10.1037/h0029780

[psyp13309-bib-0037] Kassambara , A. , & Mundt , F. (2017). factoextra: Extract and Visualize the results of multivariate data analyses. R package version 1.0.5 [Computer software]. Retrieved from https://CRAN.R-project.org/package=factoextra

[psyp13309-bib-0039] Keil, A. , Bradley, M. M. , Hauk, O. , Rockstroh, B. , Elbert, T. , & Lang, P. J. (2002). Large‐scale neural correlates of affective picture processing. Psychophysiology, 39(5), 641–649. 10.1111/1469-8986.3950641 12236331

[psyp13309-bib-0040] Kenny, P. J. (2011a). Common cellular and molecular mechanisms in obesity and drug addiction. Nature Reviews Neuroscience, 12(11), 638–651. 10.1038/nrn3105 22011680

[psyp13309-bib-0041] Kenny, P. J. (2011b). Reward mechanisms in obesity: New insights and future directions. Neuron, 69(4), 664–679. 10.1016/j.neuron.2011.02.016 21338878 PMC3057652

[psyp13309-bib-0042] Lang, P. J. , & Bradley, M. M. (2010). Emotion and the motivational brain. Biological Psychology, 84(3), 437–450. 10.1016/j.biopsycho.2009.10.007 19879918 PMC3612949

[psyp13309-bib-0043] Lang, P. J. , Bradley, M. M. , & Cuthbert, B. N. (1997). Motivated attention: Affect, activation, and action In Lang P. J., Simons R. F., & Balaban M. T. (Eds.), Attention and orienting: Sensory and motivational processes (pp. 97–135). Hillsdale, NJ: Lawrence Erlbaum Associates.

[psyp13309-bib-0044] Lang, P. J. , Bradley, M. M. , & Cuthbert, B. N. (2008). International Affective Picture System (IAPS): Affective ratings of pictures and instruction manual. Technical Report A‐8. Gainesville, FL: University of Florida.

[psyp13309-bib-0045] Littel, M. , & Franken, I. H. (2012). Electrophysiological correlates of associative learning in smokers: A higher‐order conditioning experiment. BMC Neuroscience, 13(1), 8 10.1186/1471-2202-13-8 22235938 PMC3277456

[psyp13309-bib-0046] Liu, Y. , Huang, H. , McGinnis‐Deweese, M. , Keil, A. , & Ding, M. (2012). Neural substrate of the late positive potential in emotional processing. Journal of Neuroscience, 32(42), 14563–14572. 10.1523/JNEUROSCI.3109-12.2012 23077042 PMC3516184

[psyp13309-bib-0047] Lovic, V. , Saunders, B. T. , Yager, L. M. , & Robinson, T. E. (2011). Rats prone to attribute incentive salience to reward cues are also prone to impulsive action. Behavioural Brain Research, 223(2), 255–261. 10.1016/j.bbr.2011.04.006 21507334 PMC3119757

[psyp13309-bib-0048] Mahler, S. V. , & de Wit, H. (2010). Cue‐reactors: Individual differences in cue‐induced craving after food or smoking abstinence. PLoS ONE, 5(11), 1–3. 10.1371/journal.pone.0015475 PMC2978100 21085667

[psyp13309-bib-0049] Mejova, Y. , Abbar, S. , & Haddadi, H. (2016). Fetishizing food in digital age: #foodporn around the world. ArXiv:1603.00229v2.

[psyp13309-bib-0050] Meyer, P. J. , Lovic, V. , Saunders, B. T. , Yager, L. M. , Flagel, S. B. , Morrow, J. D. , & Robinson, T. E. (2012). Quantifying individual variation in the propensity to attribute incentive salience to reward cues. PLoS ONE, 7(6), e38987 10.1371/journal.pone.0038987 22761718 PMC3382216

[psyp13309-bib-0051] Minnix, J. A. , Versace, F. , Robinson, J. D. , Lam, C. Y. , Engelmann, J. M. , Cui, Y. , … Cinciripini, P. M. (2013). The late positive potential (LPP) in response to varying types of emotional and cigarette stimuli in smokers: A content comparison. International Journal of Psychophysiology, 89, 18–25. 10.1016/j.ijpsycho.2013.04.019 23643564 PMC3771859

[psyp13309-bib-0052] Oliver, J. A. , Jentink, K. G. , Drobes, D. J. , & Evans, D. E. (2016). Smokers exhibit biased neural processing of smoking and affective images. Health Psychology, 35(8), 866–869. 10.1037/hea0000350 27505209 PMC5796754

[psyp13309-bib-0053] Olofsson, J. K. , Nordin, S. , Sequeira, H. , & Polich, J. (2008). Affective picture processing: An integrative review of ERP findings. Biological Psychology, 77(3), 247–265. 10.1016/j.biopsycho.2007.11.006 18164800 PMC2443061

[psyp13309-bib-0054] Padoa‐Schioppa, C. (2009). Range‐adapting representation of economic value in the orbitofrontal cortex. Journal of Neuroscience, 29(44), 14004–14014. 10.1523/JNEUROSCI.3751-09.2009 19890010 PMC2776749

[psyp13309-bib-0055] Patton, J. H. , Stanford, M. S. , & Barratt, E. S. (1995). Factor structure of the Barratt Impulsiveness Scale. Journal of Clinical Psychology, 51(6), 768–774. 10.1002/1097-4679(199511)51:6<768:AID-JCLP2270510607>3.0.CO;2-1 8778124

[psyp13309-bib-0056] Pessoa, L. (2009). How do emotion and motivation direct executive control? Trends in Cognitive Sciences, 13(4), 160–166. 10.1016/j.tics.2009.01.006 19285913 PMC2773442

[psyp13309-bib-0057] R Core Team . (2014). R: A language and environment for statistical computing. Vienna, Austria: R Foundation for Statistical Computing Retrieved from http://www.r-project.org/

[psyp13309-bib-0058] Robinson, J. D. , Versace, F. , Lam, C. Y. , Minnix, J. A. , Engelmann, J. M. , Cui, Y. , … Cinciripini, P. M. (2013). The CHRNA3 rs578776 variant is associated with an intrinsic reward sensitivity deficit in smokers. Frontiers in Psychiatry, 4(Sept), 25–27. 10.3389/fpsyt.2013.00114 24065931 PMC3779859

[psyp13309-bib-0059] Rousseeuw, P. J. (1987). Silhouettes: A graphical aid to the interpretation and validation of cluster analysis. Journal of Computational & Applied Mathematics, 20, 53–65. 10.1016/0377-0427(87)90125-7

[psyp13309-bib-0060] Sabatinelli, D. , Lang, P. J. , Keil, A. , & Bradley, M. M. (2007). Emotional perception: Correlation of functional MRI and event‐related potentials. Cerebral Cortex, 17(5), 1085–1091. 10.1093/cercor/bhl017 16769742

[psyp13309-bib-0061] Sarter, M. , & Phillips, K. B. (2018). The neuroscience of cognitive‐motivational styles: Sign‐ and goal‐trackers as animal models. Behavioral Neuroscience, 132(1), 1–12. 10.1037/bne0000226 29355335 PMC5881169

[psyp13309-bib-0062] Saunders, B. T. , & Robinson, T. E. (2013). Individual variation in resisting temptation: Implications for addiction. Neuroscience and Biobehavioral Reviews, 37(9), 1955–1975. 10.1016/j.neubiorev.2013.02.008 23438893 PMC3732519

[psyp13309-bib-0063] Schembre, S. , Greene, G. , & Melanson, K. (2009). Development and validation of a weight‐related eating questionnaire. Eating Behaviors, 10(2), 119–124. 10.1016/j.eatbeh.2009.03.006 19447354

[psyp13309-bib-0064] Schupp, H. T. , Cuthbert, B. N. , Bradley, M. M. , Cacioppo, J. T. , Ito, T. , & Lang, P. J. (2000). Affective picture processing: The late positive potential is modulated by motivational relevance. Psychophysiology, 37(2), 257–261. 10.1111/1469-8986.3720257 10731776

[psyp13309-bib-0065] Schupp, H. T. , Cuthbert, B. N. , Bradley, M. M. , Hillman, C. H. , Hamm, A. O. , & Lang, P. J. (2004). Brain processes in emotional perception: Motivated attention. Cognition and Emotion, 18(5), 593–611. 10.1080/02699930341000239

[psyp13309-bib-0066] Snaith, R. P. , Hamilton, M. , Morley, S. , Humayan, A. , Hargreaves, D. , & Trigwell, P. (1995). A scale for the assessment of hedonic tone. The Snaith‐Hamilton Pleasure Scale. British Journal of Psychiatry, 167, 99–103. 10.1192/bjp.167.1.99 7551619

[psyp13309-bib-0067] Tibshirani, R. , Walther, G. , & Hastie, T. (2001). Estimating the number of clusters in a data set via the gap statistic. Journal of the Royal Statistical Society. Series B (Statistical Methodology), 63(2), 411–423. Retrieved from http://www.jstor.org/stable/2680607

[psyp13309-bib-0068] Tomie, A. , Grimes, K. L. , & Pohorecky, L. A. (2008). Behavioral characteristics and neurobiological substrates shared by Pavlovian sign‐tracking and drug abuse. Brain Research Reviews, 58(1), 121–135. 10.1016/j.brainresrev.2007.12.003 18234349 PMC2582385

[psyp13309-bib-0069] Tunstall, B. J. , & Kearns, D. N. (2015). Sign‐tracking predicts increased choice of cocaine over food in rats. Behavioural Brain Research, 281, 222–228. 10.1016/j.bbr.2014.12.034 25541036 PMC4305489

[psyp13309-bib-0070] Vasey, M. W. , & Thayer, J. F. (1987). The continuing problem of false positives in repeated measures ANOVA in psychophysiology: A multivariate solution. Psychophysiology, 24(4), 479–486. 10.1111/j.1469-8986.1987.tb00324.x 3615759

[psyp13309-bib-0071] Versace, F. , Claiborne, K. N. , Deweese, M. M. , Engelmann, J. M. , Green, C. E. , Karam‐Hage, M. A. , … Cinciripini, P. M. (2017). Identifying smokers at higher risk for relapse using a newly developed neuroimaging‐based classification algorithm. Paper presented at *Conference of the Society for Research on Nicotine and Tobacco*, Florence, Italy.

[psyp13309-bib-0072] Versace, F. , Engelmann, J. M. , Deweese, M. M. , Robinson, J. D. , Green, C. E. , Lam, C. Y. , … Cinciripini, P. M. (2017). Beyond cue reactivity: Non‐drug‐related motivationally relevant stimuli are necessary to understand reactivity to drug‐related cues. Nicotine and Tobacco Research, 19(6), 663–669. 10.1093/ntr/ntx002 28486715 PMC5423099

[psyp13309-bib-0073] Versace, F. , Engelmann, J. M. , Robinson, J. D. , Jackson, E. F. , Green, C. E. , Lam, C. Y. , … Cinciripini, P. M. (2014). Prequit fMRI responses to pleasant cues and cigarette‐related cues predict smoking cessation outcome. Nicotine and Tobacco Research, 16(6), 697–708. 10.1093/ntr/ntt214 24376278 PMC4015090

[psyp13309-bib-0074] Versace, F. , Kypriotakis, G. , Basen‐Engquist, K. , & Schembre, S. M. (2016). Heterogeneity in brain reactivity to pleasant and food cues: Evidence of sign‐tracking in humans. Social Cognitive and Affective Neuroscience, 11(4), 604–611. 10.1093/scan/nsv143 26609106 PMC4814789

[psyp13309-bib-0075] Versace, F. , Lam, C. Y. , Engelmann, J. M. , Robinson, J. D. , Minnix, J. A. , Brown, V. L. , & Cinciripini, P. M. (2012). Beyond cue reactivity: Blunted brain responses to pleasant stimuli predict long‐term smoking abstinence. Addiction Biology, 17(6), 991–1000. 10.1111/j.1369-1600.2011.00372.x 21967530 PMC3252422

[psyp13309-bib-0076] Versace, F. , Minnix, J. A. , Robinson, J. D. , Lam, C. Y. , Brown, V. L. , & Cinciripini, P. M. (2011). Brain reactivity to emotional, neutral and cigarette‐related stimuli in smokers. Addiction Biology, 16(2), 296–307. 10.1111/j.1369-1600.2010.00273.x 21182573 PMC3058803

[psyp13309-bib-0077] Versace, F. , & Schembre, S. M. (2015). “Obesogenic” oversimplification. Obesity Reviews, 16(8), 702–703. 10.1111/obr.12301 26179234

[psyp13309-bib-0078] Volkow, N. D. , Wang, G. J. , Tomasi, D. , & Baler, R. D. (2013). Obesity and addiction: Neurobiological overlaps. Obesity Reviews, 14(1), 2–18. 10.1111/j.1467-789X.2012.01031.x 23016694 PMC4827343

[psyp13309-bib-0079] Watson, D. , Clark, L. A. , & Tellegen, A. (1988). Development and validation of brief measures of positive and negative affect: The PANAS Scales. Journal of Personality and Social Psychology, 54, 1063–1070. 10.1037/0022-3514.54.6.1063 3397865

[psyp13309-bib-0080] Weinberg, A. , & Hajcak, G. (2010). Beyond good and evil: The time‐course of neural activity elicited by specific picture content. Emotion, 10(6), 767–782. 10.1037/a0020242 21058848

[psyp13309-bib-0081] Yager, L. M. , & Robinson, T. E. (2010). Cue‐induced reinstatement of food seeking in rats that differ in their propensity to attribute incentive salience to food cues. Behavioural Brain Research, 214(1), 30–34. 10.1016/j.bbr.2010.04.021 20416342 PMC2910199

