# Supplementary material for: The reality of “food porn”: Larger brain responses to food‐related cues than to erotic images predict cue‐induced eating
Source: Psychophysiology. 2018 Dec 16;56(4):e13309. doi: 10.1111/psyp.13309 (PMC6446735; doi:10.1111/psyp.13309)
Supplement: Supplementary file 1 — Figure S1 Figure S2 Table S1 Table S2 Table S3 [file PSYP-56-na-s001.docx]

**The reality of “food porn”: Larger brain responses to food-related than erotic images predict cue-induced eating**

Francesco Versace, David W. Frank, Elise M. Stevens, Menton M. Deweese, Michele Guindani, Susan M. Schembre

The University of Texas MD Anderson Cancer Center

**Supplementary Results**

**Demographics and Self-report Questionnaires**

**Table S1** shows demographic and self-report variables separated by cluster allocation. All the analyses conducted on the demographics and self-report questionnaires were not corrected for multiple comparisons. The two groups did not differ in terms of gender distribution (*P*=.18), age (*P*=.35), or BMI (*P*=.74). On the Barratt Impulsivity scale (BIS), the total score suggested that individuals classified as C>P were somewhat more impulsive than individuals classified at P>C, but the differences were not statistically significant (*P*=.15). The analyses conducted on the BIS subscales showed that individuals classified as C>P reported significantly (*P*<.05) higher scores than individuals classified as P>C in the attentional impulsiveness subscale (i.e., “focusing on the task at hand” and “thought insertion and racing thoughts”) and the non-planning impulsiveness subscale (i.e., “planning and thinking carefully” and “enjoying challenging mental tasks”), but not on the motor impulsiveness subscale (i.e., “acting on the spur of the moment” and “a consistent life style”). Mood questionnaires (CES-D, PANAS, SHAPS) did not show any significant difference between groups (all *Ps*>.18). The WREQ total score and the scores to its subscales were similar in the two groups (all *Ps*>.27). The level of satiety in the two groups was similar before (*P*>.44) and after the session (*P*>.49).

**Table S1**

**Participant demographic information and questionnaire scores by cluster membership**

| Characteristic | All  (n=49) | C>P  (n=20) | P>C  (n=29) | P-value |
| --- | --- | --- | --- | --- |
| Age (years) | 47 | 46 | 48 | .46 |
| Women | 45% | 35% | 51% | .25 |
| Race |  |  |  |  |
| African American | 67% | 75% | 62% |  |
| Caucasian | 26% | 20% | 31% |  |
| Other | 7% | 5% | 7% |  |
| BMI | 31 | 31 | 31 |  |
| BIS |  |  |  |  |
| Attentional | 14.16 | 15.75 | 13.07 | .01 |
| Motor | 21.49 | 22.00 | 21.14 | .49 |
| Non planning | 23.06 | 25.15 | 21.62 | .04 |
| CESD | 7.20 | 8.25 | 6.48 | .24 |
| SHAPS | 47.55 | 48.15 | 47.14 | .53 |
| PANAS + | 34.63 | 35.35 | 34.14 | .65 |
| PANAS - | 17.39 | 19.10 | 16.21 | .18 |
| WREQ |  |  |  |  |
| Routine Restraint | 1.76 | 1.58 | 1.88 | .27 |
| Compensatory Restraint | 2.09 | 1.98 | 2.16 | .33 |
| Susceptibility to External Cues | 1.94 | 2.09 | 1.83 | .55 |
| Emotional Eating | 1.60 | 1.64 | 1.57 | .72 |
| SLIM (pre) | -4.43 | -8.97 | -1.29 | *.45* |
| SLIM (post) | -10.15 | -14.88 | -6.89 | *.50* |

NOTE: P-values estimated by independent t-tests or chi-square analyses. C>P = individuals with larger LPPs to food-predictive cues than erotic stimuli; P>C = individuals with larger LPPs to erotic stimuli than food-predictive cues; BMI = Body Mass Index; BIS = Barratt Impulsiveness Scale; CESD = The Center for Epidemiological Studies Depression Scale; SHAPS = Snaith-Hamilton Pleasure Scale; PANAS = Positive and Negative Affect Schedule; WREQ = Weight-Related Eating Questionnaire; SLIM = Satiety Labeled Intensity Magnitude.

**Cluster analysis**

**Figure S1 and S2** show the results of the silhouette method and the gap statistic method to choose the optimal number of clusters on the k-means clustering algorithm, as implemented in the R module “factoextra” (Kassambara & Mundt, 2017). Both criteria indicated that the two-cluster solution is the most appropriate.

**Figure S1**


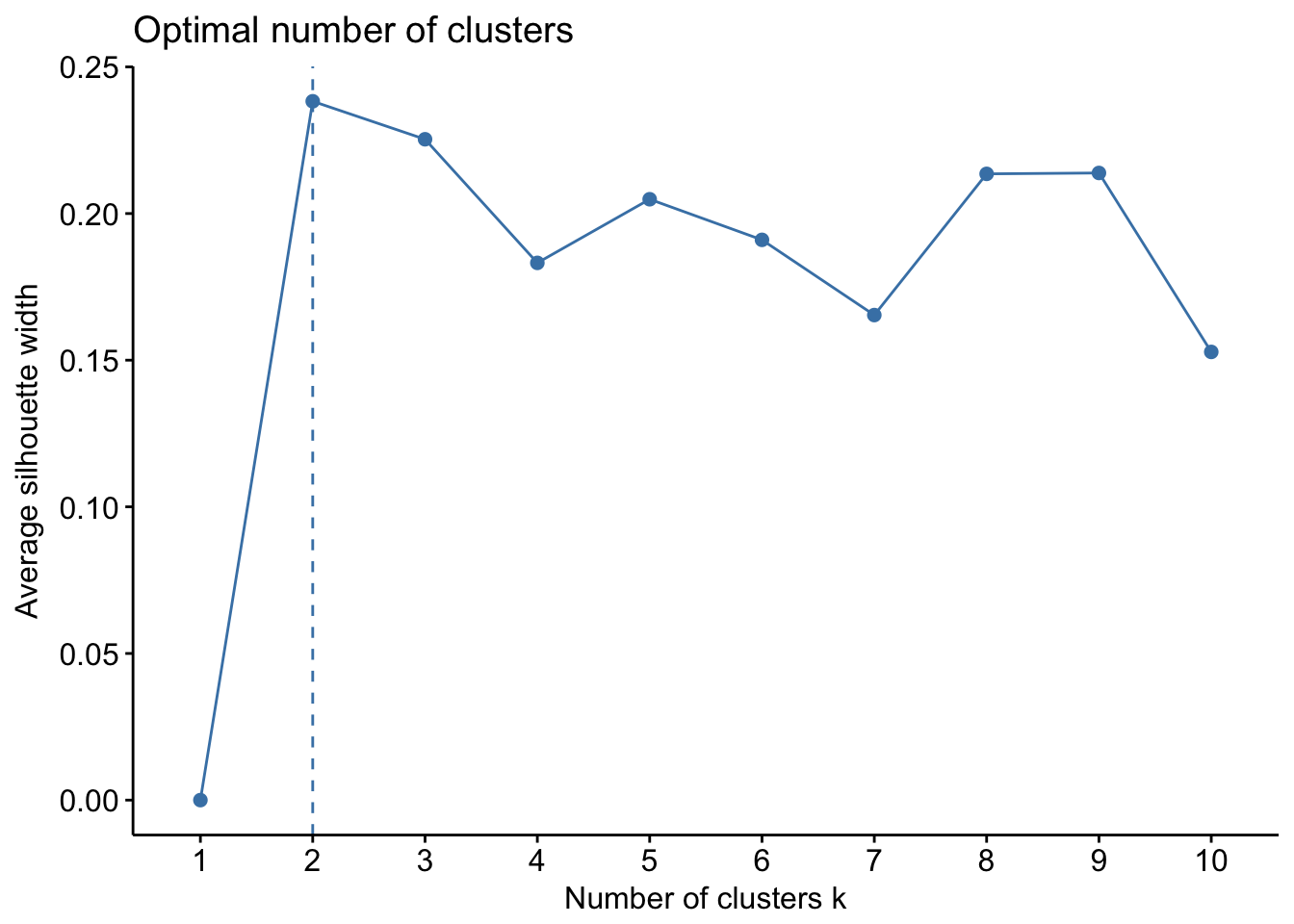


The silhouette method indicated that two was the optimal number of clusters identified by the *k*-means cluster analysis using the 8 LPP values.

**Figure S2**


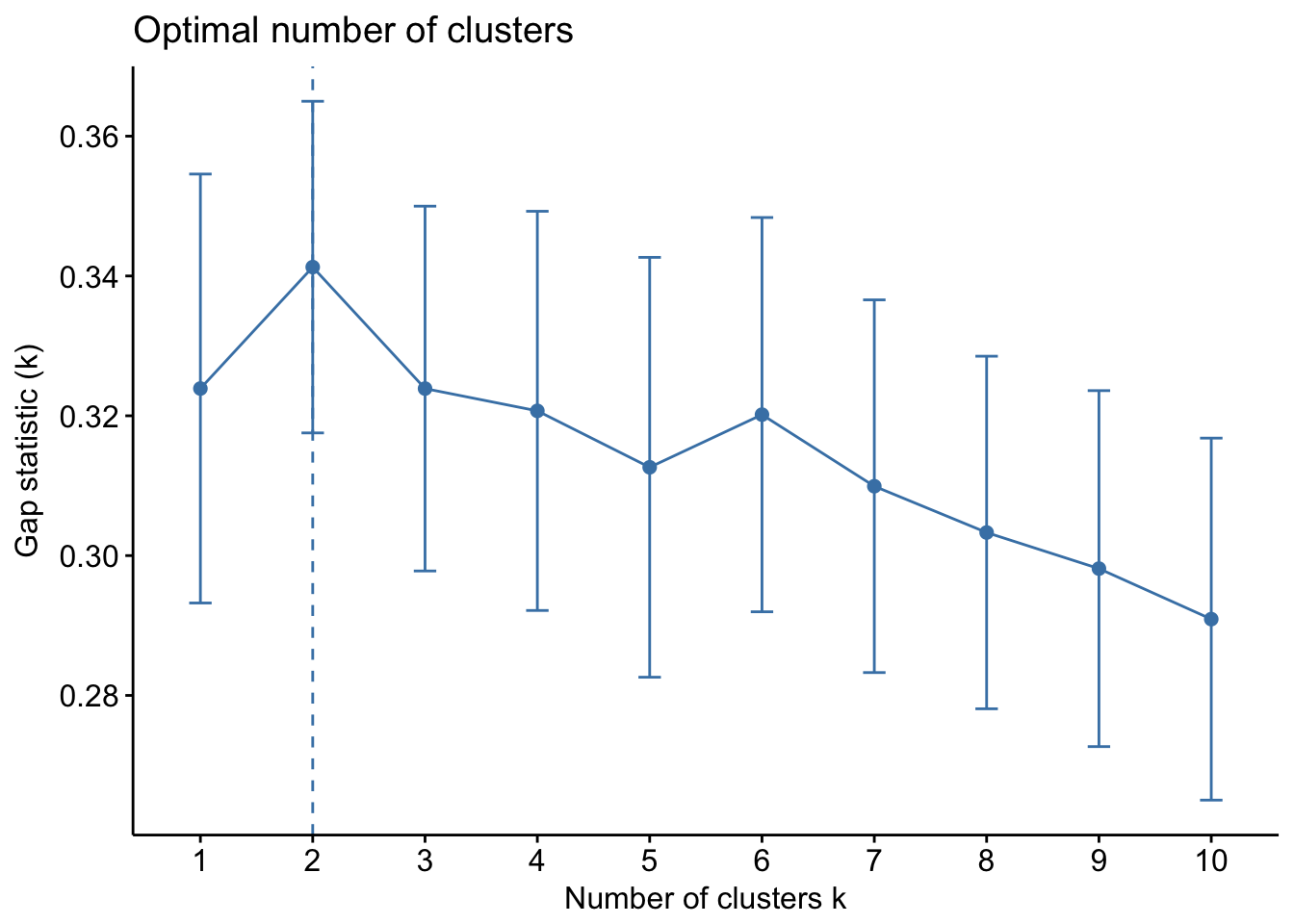


The gap statistic method indicated that two was the optimal number of clusters identified by the *k*-means cluster analysis using the 8 LPP values.

**Analysis of eating behavior patterns and brain reactivity**

**Table S2** shows the results of an unadjusted quasi Poisson generalized linear regression model (quasi-Poisson GLM) relating the number of candies eaten by the participants to their cluster assignment. The quasi Poisson GLM relies on a log link to relate the regression equation to the count response, by positing $E\left( Y | X \right)=\mu, log\left( \mu\right)=X^{'}\beta.$The coefficients of the regression identify changes in the response rate for a unit-increase in the corresponding covariate on the log-scale, with respect to the reference baseline. **Table S3** shows the results of the quasi-Poisson GLM when adjusting for known potential confounders (age, gender, BMI and level of appetite at the beginning of the experiment).

**Table S2**

The Unadjusted quasi Poisson Regression Analysis showed a significant (*P*=.01) difference in eating behavior by cluster allocation.

| **term** | **estimate** | **std.error** | **statistic** | ***P* value** |
| --- | --- | --- | --- | --- |
| Intercept | 2.130 | 0.255 | 8.338 | 0.00 |
| Cluster Allocation  (baseline: C>P) | 0.868 | 0.324 | 2.680 | 0.01 |

**Table S3**

The quasi-Poisson Regression Analysis showed the presence of significant (*P*=.024) differences in eating behavior by cluster allocation after adjusting for age, gender, BMI, and pre-experiment lever of appetite.

| **term** | **estimate** | **std.error** | **statistic** | ***P* value** |
| --- | --- | --- | --- | --- |
| Intercept | 3.417 | 0.819 | 4.170 | 0.000 |
| Cluster Allocation  (baseline: C>P) | 0.802 | 0.343 | 2.337 | 0.024 |
| Age | -0.031 | 0.014 | -2.156 | 0.037 |
| Gender | -0.283 | 0.346 | -0.819 | 0.417 |
| BMI | 0.008 | 0.018 | 0.460 | 0.648 |
| Pre-experiment Level of Appetite | -0.003 | 0.005 | -0.594 | 0.555 |

**References**

Kassambara, A., & Mundt, F. (2017). factoextra: Extract and Visualize the Results of Multivariate Data Analyses. R package version 1.0.4. Retrieved from http://www.sthda.com/english/rpkgs/factoextra
